# Serovars, virulence factors, and antimicrobial resistance profile of non-typhoidal *Salmonella* in the human-dairy interface in Northwest Ethiopia: A one health approach

**DOI:** 10.1371/journal.pntd.0012646

**Published:** 2024-11-20

**Authors:** Achenef Melaku Beyene, Yismaw Alemie, Mucheye Gizachew, Ahmed E. Yousef, Bereket Dessalegn, Abebe Belete Bitew, Amare Alemu, Waktole Gobena, Kornschober Christian, Baye Gelaw

**Affiliations:** 1 Department of Medical Microbiology, College of Medicine and Health Sciences, University of Gondar, Gondar, Ethiopia; 2 Department of Pathobiology, College of Veterinary Medicine and Animal Sciences, University of Gondar, Gondar, Ethiopia; 3 Department of Food Science and Technology, The Ohio State University, Columbus, Ohio, United States of America; 4 Department of Veterinary Epidemiology and Public Health, College of Veterinary Medicine and Animal Sciences, University of Gondar, Gondar, Ethiopia; 5 Infectious Diseases Directorate, Tuberculosis and Other Bacterial Diseases division, Ethiopian Public Health Institute, Addis Ababa, Ethiopia; 6 Food Microbiology Laboratory, Ethiopian Public Health Institute, Addis Ababa, Ethiopia; 7 AGES: Agency for Health and Food Safety, National Reference Laboratory for *Salmonella*, Institute for Medical Microbiology and Hygiene, Graz, Austria; University of Oxford, UNITED KINGDOM OF GREAT BRITAIN AND NORTHERN IRELAND

## Abstract

Non-typhoidal *Salmonella* (NTS) is a zoonotic pathogen that exerts huge public health and economic impacts in the world. The severity of illness is mainly related to the serovars involved, the presence of virulence genes, and antimicrobial resistance (AMR) patterns. However, data are scarce on serovars, virulence genes, and AMR among NTS identified from the human-dairy interface in Northwest Ethiopia. Thus, this study investigated the serovars, common virulence genes, and AMR patterns of NTS isolates in the area. The study was conducted from June 2022 to August 2023 among randomly selected 58 dairy farms. A total of 362 samples were processed to detect NTS using standard bacteriological methods. The presumptive positive colonies were confirmed by Matrix-Assisted Laser Desorption Ionization-Time-of-Flight (MALDi-ToF). Polymerase chain reaction (PCR) was used to detect virulence genes, including *invA* and *spv*C. A slide agglutination test according to the White-Kauffmann-Le Minor scheme was employed to identify the serovars of the NTS isolates. The Kirby-Bauer disk diffusion method was used to assess the antimicrobial susceptibility patterns. Of the processed samples (362), 28 (7.7%) NTS isolates were detected. When distributed among samples, the proportions were 11.9%, 10.5%, 10.3%, 5.2%, 4.3%, and 1.7% among cows’ feces, dairy farm sewage, pooled raw milk, milk container swabs, milkers’ stool, and milkers’ hand swab samples, respectively. Six serovars were detected with the dominancy of *S*. Uganda (39.3%), followed by *S*. *enterica* subsp. *diarizonae* (25.0%) and *S*. Typhimurium (21.4%). Among the 28 NTS isolates, 100% and 21.4% had the virulence genes *invA* and *spv*C, respectively. The susceptibility profile showed that 89.3% of the NTS isolates were resistant to at least one antimicrobial agent and 46.4% were resistant to three or more classes of antimicrobials (multidrug-resistant). Among antimicrobials, isolates were highly resistant to ampicillin (57.1%), followed by tetracycline (42.9%) and chloramphenicol (35.7%). On the other hand, the NTS isolates were 100%, 96.4%, and 96.4% susceptible to ceftriaxone, azithromycin, and norfloxacin, respectively. In conclusion, we detected NTS from humans, dairy cows, raw milk, dairy utensils, and the environment (sewage), showing the potential of the human-dairy farm-environment nexus in the NTS circulation. These further highlight that the interface is a good point of intervention in the control and prevention of NTS infection. The susceptibility profiles of the isolate necessitate interventions including the prudent use of the antimicrobials.

## Author summary

Non-typhoidal *Salmonella* (NTS) is a bacterium that induces mild to severe life-threatening syndromes in humans and animals worldwide. The severity of NTS is affected by the serovars involved, the presence of the virulence genes, and the ability to resist antimicrobials. In this study, we followed an integrated approach involving three key sectors (humans, the food chain (food), animals, and the environment) (one-health approach), which is highly recommended in areas where their interaction is high (dairy farms). This study confirmed that the pathogen was circulating in the human-dairy interface. Six serovars were detected, which were dominated by *S*. Uganda, *S*. *enterica* subsp. diarizonae, and *S*. Typhimurium. Of the isolates, 89.3% were resistant to at least one antimicrobial and 46.4% were multidrug-resistant. Hence, our research confirmed that the human-dairy interface is one source of future outbreaks of NTS. The serovars, virulent genes, and resistant character of isolates are suggestive of the tendency of the infection to become more severe and the potential to induce high mortality in the community. Therefore, intervention measures have to be implemented.

## Introduction

*Salmonella enterica* is a Gram-negative bacterial pathogen that consists of more than 2,600 serovars [1,2]. Some of these serovars are host-restricted like typhoidal serovars, and the majority of the serovars are host-unrestricted (non-typhoidal) [3]. The non-typhoidal serovars have wider host ranges, are more common, easily transmissible, hard to control, and contribute to a significant proportion of Salmonellosis in humans and animals worldwide [4,5]. It has been estimated that non-typhoidal *Salmonella* (NTS) causes about 93.8 million illnesses and 230 thousand fatalities in humans per year globally [6]. In 2017, the invasive form of NTS caused about 535,000 illnesses and 77,500 deaths, being highest in Sub-Saharan Africa and vulnerable groups of the population (children, elderly people, and people with HIV infection) [7,8].

The syndromes due to the infections of NTS may range from mild gastroenteritis to severe, life-threatening systemic illnesses. The severity or burden of the infection in patients is highly related to the presence of virulence factors in the pathogen [9,10] and the serovar involved [9,11]. Virulence agents (encoding genes) such as invasion A (*invA*) and plasmid virulence (*spvC*) are important factors in the invasion of host epithelial cells and systemic illnesses, respectively [12,13]. The serovars of NTS can be identified using antigenic variations in the O (lipopolysaccharide), H (flagella), and Vi (capsular) antigens by the White-Kauffmann-Le Minor scheme [14,15]. Determining the NTS serovars is very important to understand the clinical manifestations, environmental adaptability, antimicrobial susceptibility, and other characteristics of the pathogen [16].

The emergence of antimicrobial resistance (AMR) among NTS serovars is becoming a public health concern in the world. The development of AMR is highly related to the overuse and misuse of antimicrobials, which creates a selective pressure so that the susceptible group diminishes and resistant ones flourish [2,17].

The majority of NTS infections in humans are associated with the consumption of contaminated food, unprotected contact with infected animals, and a contaminated environment. Cattle are an important reservoir of NTS that plays a role in the contamination of foods from animal sources like meat and milk and the environment [18]. The frequent interaction of humans, animals, and the environment in the human-dairy interface may be attributed to NTS outbreaks in the community [18,19].

The NTS is a significant problem in developing countries like Ethiopia due to unhygienic practices, lack of infrastructure needed for proper implementation of food safety measures and laws, weak regulatory systems, a habit of consuming raw or inadequately cooked food items, and frequent and unprotected contact with animals and the environment [20–22]. It is one of the top ten disease-causing agents ranked based on the impact on the intensification of animal production [23]. Assessing the occurrence of NTS and characterization will help to devise evidence-based control and prevention measures that can reduce the risks of infection. The prevalence of NTS in Ethiopia has been reported in both humans and animals [20,24,25]. However, in the Northwest part of Ethiopia, such information is limited or not well documented. Furthermore, the prevalence of NTS serovars and the presence of common virulence factors haven’t been well characterized in the area. Therefore, this study was conducted to detect NTS and determine the circulating serovars, common virulence genes, and antimicrobial resistance patterns of isolates in the human-dairy interface in Northwest Ethiopia by following a one-health approach.

## Materials and methods

### Ethical approval

Ethical approval was obtained from the University of Gondar’s ethical review board with reference number VP/RTT/05/60/2021 for its ethical soundness and acceptability. The letters of introduction and cooperation were written from the University of Gondar to the selected districts. At the farm level, the farm owners and study participants in dairy farms were informed about the purpose and procedures of the study. Verbal informed consent was obtained from each human participant in the selected dairy farms.

### Study area

**Fig 1:**
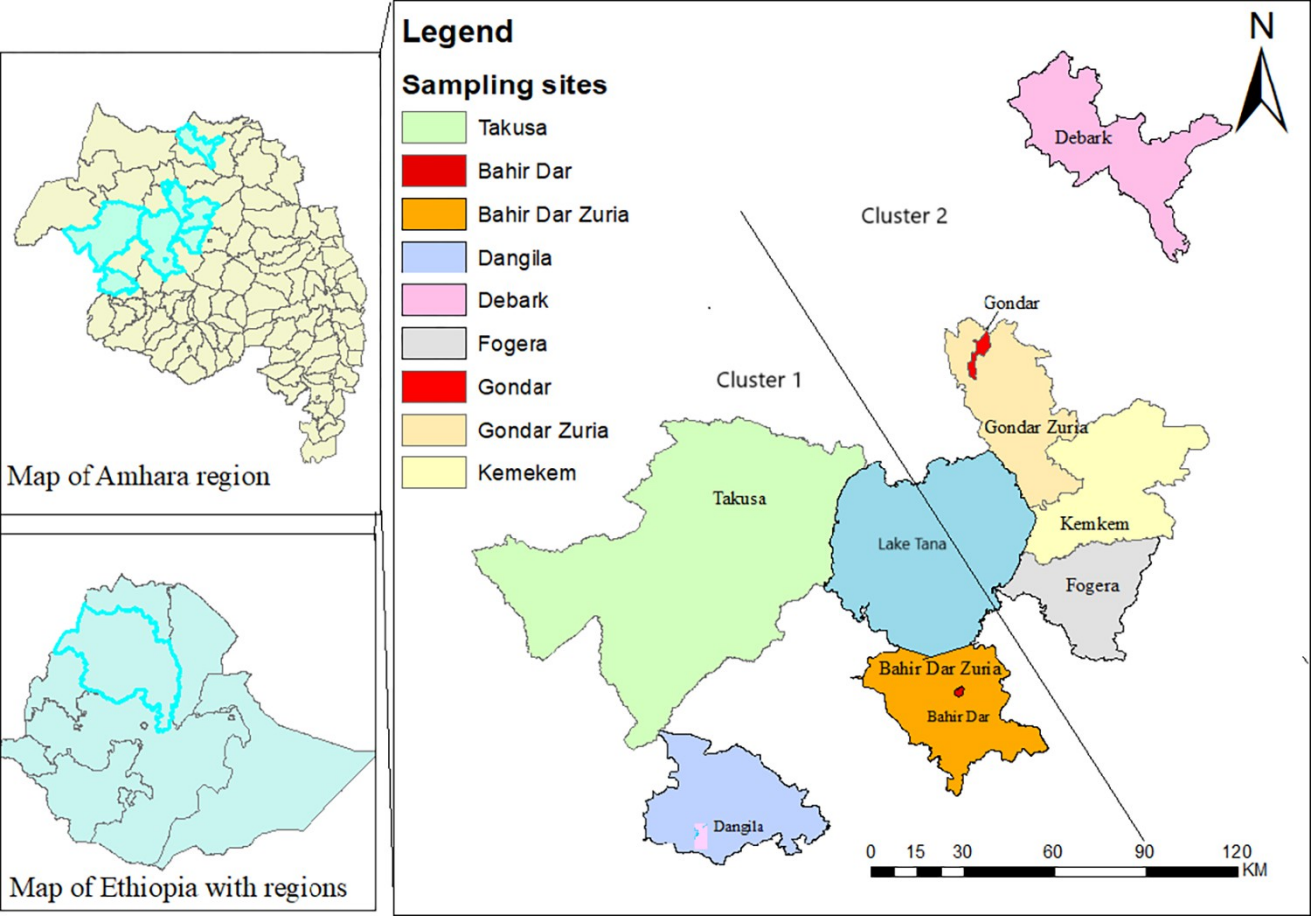
Map of the study area (sketched by ArcGIS maps; the sampling sites were divided into two clusters by the solid line based on their geographic proximity). The shape files were downloaded from https://diva-gis.org/data.html.

The study was conducted in the northwest part of Ethiopia. In the area, two cities (Bahir Dar and Gondar), and seven districts (Dangila, Fogera, Kemkem, Takusa, Bahir Dar Zuria, Gondar Zuria, and Debark) were selected based on their relatively higher dairy production activities (Fig 1). These areas have elevations ranging from 1820 to 2706 meters above sea level and are located between 10° 30’ 0" North (N) and 12° 0’ 0" N latitude, and between 36° 43’ 12" East (E) and 38° 4’ 48" E longitude. The average annual temperature and rainfall range from 10 to 30°C and 1,000 to 1,500 mm, respectively [26]. The area consists of small to medium-sized dairy farms. Dairy farmers in this area supply milk and milk products to urban centers in the Amhara regional state or beyond, where dairy product consumption is high [27].

### Study design and study population

The study was cross-sectional and conducted from June 2022 to August 2023 by targeting raw milk, dairy cows, milk containers, contact persons (milkers), and the environment among dairy farms in selected sites. All humans and animals sampled were apparently healthy and were not taking antimicrobials at the time of sampling. All animals were lactating cows. Based on the number of animals within the farm, the dairy farms were classified as small (20 or fewer animals), medium, or above (21 or more animals) dairy farms [18]. Cow’s body condition was categorized as poor, medium, and good based on the assessment of its condition at different directions and visibility of bones as described by Mishra *et al*. [28].

### Sample size estimation and sampling

The sample size was estimated by considering a 5% margin of error, at a 95% confidence interval and the 19.4% maximum reported prevalence of NTS in Ethiopia [29,30]. Accordingly, the minimum calculated sample size was 241. However, a total of 362 samples were collected to increase the representativeness due to multiple sample types in a selected farm [31,32] and the interest in isolating other bacteria from the samples. In the selected sampling sites, the dairy farms were randomly selected from the list of dairy farms in each district’s livestock development offices and included in the study. The number of dairy farms enrolled in each study site was determined proportionally based on the total number of dairy farms in each site [33]. From each selected dairy farm, at least six types of samples (pooled raw milk, stool from milkers, dairy farm sewage, feces from lactating cows, swabs from milk containers, and milkers’ hands) were collected. If the number of dairy cows on the farm was greater than 10, two fecal samples from lactating cows were collected.

### Sample collection and transportation

Information related to the milkers’ socio-demographic characteristics, farm size, milkers and milk containers as well as feed and water source of dairy cows were collected by using a personal observation, checklist and interviewing the milkers. Samples were collected by using sterile containers. Approximately 50 ml of pooled raw milk were collected aseptically. Swabs from milkers’ hands and milk containers were collected separately by using sterile cotton swabs and placed into test tubes containing sterile buffered peptone water (BPW) (Granucult, Germany). About 10 ml of effluent samples were collected and placed into sterile containers. Similarly, about 10 grams of stool samples were collected in sterile stool cups with applicator sticks from milkers. Finally, approximately 10 grams of fecal samples were collected directly from the rectum of randomly selected lactating dairy cows using sterile disposable gloves and added into the sterile screw-capped tube. Samples were transported using an icebox to the food safety laboratory of the University of Gondar, Ethiopia. On arrival, samples were processed immediately or put in a refrigerator (~4°C) and processed within 24 hours of collection.

### Bacteriological analysis

The isolation of NTS was performed using the techniques recommended by the International Organization for Standardization [34]. Each sample type was first enriched non-selectively in BPW at a 1:10 ratio. Swab samples transported in 9 ml of BPW were incubated directly. Three grams of animal feces, human stool, and sewage samples were separately transferred into a sterile container with 27 ml of sterile BPW. Twenty-five ml of the milk sample was transferred into a sterile screw-capped glass bottle containing sterile 225 ml BPW. The sample with enrichment fluid was mixed thoroughly and incubated at 37°C for 24 hours (S3 Fig).

The Rappaport-Vassiliadis soya (RVS) broth (Oxoid, England) and Muller-Kauffmann Tetrathionate-Novobiocin (MKTTn) broth (Oxoid, UK) were used for selective enrichment. From the enriched fluid, 0.1 ml was transferred into a tube containing 10 ml of RVS broth and incubated at 41.5°C for 24 hours. Another 1 ml of the enriched sample was transferred into a tube containing 10 ml of MKTTn broth and incubated at 37°C for 24 hours. Then, a loop full of enriched sample was plated on xylose lysine deoxycholate (XLD) agar (Hi-Media, India) and Hektoen enteric (HE) agar (Thermofisher, Netherlands). After incubation, the plates were examined for the presence of typical colonies (pink colonies with or without black centers on XLD agar and blue-green to blue colonies with or without black centers on HE agar) [34] (S1 Fig). From both selective plating mediums, three to five suspected colonies were taken and streaked separately over the surface of tryptone soya agar (Sigma-Aldrich, USA), and then incubated for 24 hours at 37°C for biochemical characterization. Colonies on tryptone soya agar were subjected to preliminary biochemical screening using urease, triple sugar iron agar, lysine decarboxylase, and indole tests. All presumptive NTS isolates that fulfilled the preliminary biochemical tests were further characterized by the analytical profile index (API) 20E test (BioMerieux Inc. France). All NTS isolates that passed the API 20E test were confirmed by Matrix-Assisted Laser Desorption Ionization–Time-of-Flight (MALDI-TOF) (S3 Fig).

### Serovar identification

The serovar identification of NTS isolates was conducted by slide agglutination test using a *Salmonella* serotyping kit (SSI, Austria) according to the White-Kauffmann-Le Minor scheme [15,35]. The laboratory procedure was performed at the National Reference Laboratory for *Salmonella*, Graz, Austria.

### Detection of common virulence genes

The DNA of the bacterial isolates was extracted using the heat lysis method [36]. Briefly, 250 μl of the overnight incubated broth was centrifuged at 11,000 rpm for 3 minutes. The supernatant was discarded and the pellets were washed with 1000 μl sterile physiological saline (0.85%). Subsequently, 100 μl of nuclease-free water was added to the pellets, and the mixture was mixed by vortex mixer, boiled at 100°C for 10 minutes, then chilled on ice for about 5 minutes. Finally, the cell debris was separated by centrifugation at 13,500 rpm for 5 minutes and the supernatant was taken and stored at -20°C until use as the DNA template.

Amplification of the gene *invA* was carried out in a total volume of 20 μl containing 10 μl of the Master mix (2x GoTaq colorless Master mix, Promega USA), 0.62 μl of 10 μM forward primer, 0.65 μl of 10 μM reverse primer, 6.73 μl nuclease-free water and 2 μl DNA template [37]. The reaction mixture for amplification of *spvC* gene was prepared similarly, except using 0.8 μl forward and 0.8 μl reverse primer and 6.4 μl nuclease-free water [38]. The PCR thermal cycling conditions, primers, and amplicon lengths for the target genes are listed in Table 1. The initial denaturation, total cycle, and final extension in all reactions were 95°C for 5 minutes, 30, and 72°C for 5 minutes, respectively [38,39].

**Table 1:** Primers, amplicon length, and PCR conditions used to screen the two *Salmonella* virulence genes.

Target gene	Primer sequence	Amplicon size (bp)	References	PCR conditions		
				Denaturation	Annealing	Extension
*inv*A	F 5’-GCTGCGCGCGAACGGCGAAG	389	[37]	95°C for 60 sec	62°C for 40 sec	72°C for 50 sec
	R 5’-TCCCGGCAGAGTTCCCATT					
*spv*C	F 5’-ACTCCTTGCACAACCAAATGCGGA	571	[39]	95°C for 60 sec	62°C for 40 sec	72°C for 60 sec
	R 5’-TGTCTTCTGCATTTCGCCACCATCA					

### Antimicrobial susceptibility test

For the antimicrobial susceptibility test, the Kirby-Bauer disk diffusion method was used. Each bacterial isolate was tested for a set of 13 antimicrobials, which are grouped into 8 classes. Each isolated NTS was inoculated on an agar plate and allowed to grow overnight at 37°C. Colonies of the isolates were emulsified in physiological saline (0.85% NaCl) solution to the concentration equivalent to a turbidity of 0.5 McFarland standards. The bacterial suspensions were inoculated on Mueller-Hinton agar (Oxoid, England) by using a sterile cotton swab. A paper disk impregnated with a fixed concentration of antimicrobial agent was applied and incubated for 24 hours. After incubation, the diameter of the zone of inhibition was measured using a caliper and classified as “sensitive,” “intermediate,” and “resistant” based on the Clinical Laboratory Standard Institute [40] guideline (S1 Table).

### Quality assurance

All media were prepared based on the manufacturers’ recommendation, and the sterility of each batch was checked before inoculation and testing. *Salmonella* Typhimurium ATCC 13311 was used as a positive control during biochemical and molecular tests.

### Data management and analysis

The data were recorded and coded using a Microsoft Excel spreadsheet and summarized using descriptive statistics. The bivariate logistic regression was used to determine the association of NTS isolates across different variables using SPSS software version 20. The association with a *p*-value of ≤ 0.05 was considered statistically significant. The percentage of serovars, virulence genes, and AMR were calculated from the total confirmed NTS isolates.

## Results

### Demography and characteristics of sample sources

A total of 362 samples were collected and analyzed. As indicated in Table 2, most of the milkers were male (75.9%), aged from 31 to 50 years (53.4%), and had primary or basic educational levels (29.3%). Most of the farms were small-sized (82.8%), with zero grazing (87.9%), used tap water as the primary water source (77.6%), and used plastic non-food grade containers (58.6%) for milking and storage. Most of the sampled cows were aged between 6–10 years (63.1%) and medium in body condition (56.0%). Milking was conducted by hand in all farms.

**Table 2:** The prevalence of non-typhoidal *Salmonella* in different categories of human, animal, and farm.

Group	Factors	Categories	No examined	No positive	Percent positive	*p-*value	*COR*	*95% CI*
Milker’s sampled*	Sex	Female	14	0	0.0	-	-	-
		Male	44	3	6.8			
	Age	18–30 years	19	3	15.8	-	-	-
		31–50 years	31	0	0.0			
		>50 years	8	0	0.0			
	Education	Illiterate (cannot write or read)	15	0	0.0	-	-	-
		Primary or basic	17	0	0.0			
		Secondary	13	2	15.4			
		Tertiary (diploma or above)	13	1	7.7			
Dairy Farms	Size	Small (≤ 20 animals)	48	17	35.4	0.43	0.56	0.14–2.32
		Medium or large (≥21 animals)	10	6	60.0		Reference	
	Feed source	Free grazing (off-farm)	7	4	57.1	0.17	4.64	0.51–42.25
		Zero grazing (in-farm)	51	19	37.3	Reference		
	Primary water source	Tap water	45	17	37.8	0.38	0.5	0.1–2.39
		Well or underground water	8	4	50.0	0.72	1.5	0.16–14.42
		River or lake water	5	2	40.0	Reference		
	Commonly used milking container	Aluminium or nickel	6	3	50.0	0.99	0.00	
		Plastic food grade	6	4	66.7	0.92	0.89	0.09–9.16
		Plastic non-food grade	34	11	32.4	0.92	1.08	0.21–5.73
		Traditional (gourd)	12	5	41.7	Reference		
Animals (dairy cows)*#	Age	3–5 years	17	1	5.9	0.44	0.37	0.03–4.63
		6–10 years	53	7	13.2	0.92	0.91	0.17–4.97
		>10 years	14	2	14.3	Reference		
	Body condition	Good	23	0	0.0	0.99	0.00	-
		Medium	47	7	14.9	0.56	0.64	0.14–2.90
		Poor	14	3	21.4	Reference		

COR, crude odds ratio; CI, Confidence Interval

*Both humans and animals sampled were apparently healthy and were not taking antimicrobials at the time of sampling

#All animals were female (lactating cows), due to a low number of positive samples, it was impossible to conduct binary logistic regression among human factors (S1 Data).

### Detection of Non-typhoidal *Salmonella*

The overall proportion of NTS was 7.7% with a 95% confidence interval of 5.4–11.0%. The highest proportion of NTS was observed in cows’ feces (11.9%), whereas the lowest was in the milker’s hand swab samples (1.7%) (Table 3). Table 3 also depicts the proportion of NTS detected in different sampling sites. The highest proportion was on dairy farms found in Takusa (14.3%), and the lowest was among samples collected in the Dangila district (4.5%). These areas were clustered based on their geographic proximity and potential contacts, and compared. However, a difference was not observed between the two clusters.

**Table 3:** The proportion of non-typhoidal *Salmonella* isolates based on sample types and sampling sites.

The character of the Samples	Categories	Number examined	Number positive		Percent
Sample type	Cow’s feces	84	10	11.9	
	Dairy farm sewage	57	6	10.5	
	Pooled raw milk	58	6	10.3	
	Milk container swabs	58	3	5.2	
	Milker’s stool	47	2	4.3	
	Milker’s hand swabs	58	1	1.7	
Sampling sites	Takusa	35	5	14.3	
	Fogera	36	4	11.1	
	Bahir Dar	57	6	10.5	
	Gondar	44	3	6.8	
	Gondar Zuria	37	2	5.4	
	Debark	38	2	5.3	
	Kemkem	36	2	5.6	
	Dangila	44	2	4.5	
	Bahir Dar Zuria	35	2	5.2	
Sampling site clusters*	Cluster 1	171	15	8.8	
	Cluster 2	191	13	6.8	
Total		362	28	7.7	

(The sampling sites were clustered based on geographical proximity and potential contacts; cluster 1: Takusa, Bahir Dar, Dangila, Bahir Dar Zuria; cluster 2: Gondar, Gondar Zuria, Debark, Kemkem, Fogera (Fig 1 and S1 Data).

Among the human samples (n = 58) (stool and/or hand swabs), 3 (5.2%) NTS isolates were detected, and all were isolated from males, and ages ranged from 18 to 30 years. Among the 58 dairy farms, 23 (39.7%) had at least one positive sample. The detection proportions were not statistically significant among herd size, feed sources, primary water sources, and commonly used milking container types of farms. Among the samples collected from lactating cows (feces) (n = 84), 10 (11.9%) were positive for NTS. The data showed that there was no statistically significant association between the occurrence of NTS with both age and body condition categories of the cows (Table 2).

### Serovars of non-typhoidal *Salmonella* isolates

Six serovars were detected, which belonged to two subspecies (*S*. *enterica* subspecies *enterica* (75.0%) and *S*. *enterica* subspecies *diarizonae* (25.0%). The dominant serovars were *S*. Uganda (39.3%), *S*. *enterica* subsp. *diarizonae* (25.0%), and *S*. Typhimurium (21.4%). The isolates have 8 antigen formulas (Table 4). Table 4 also shows the serovars in different sample types. The dominant serovar (*S*. Uganda) was isolated from pooled raw milk, milk containers, sewage, and cow’s fecal samples.

**Table 4:** Serovar of non-typhoidal *Salmonella* isolates in different sample types.

Serovars	Antigen formula	Overall proportion N = 28 (n (%))	Sample types					
			Pooled raw milk (n (%))	Milk container swabs (n (%))	Milker’s hand swab (n (%))	Dairy farm sewage (n (%))	Human stool (n (%))	Cows’ feces (n (%))
*S*. Uganda	3,10: l,z13: 1,5	11 (39.3)	4 (36.4)	1 (9.1)	0 (0.0)	3 (27.3)	0 (0.0)	3 (27.3)
*S*. *enterica* subsp. *diarizonae*	61: c: z35 and 47: i: z	7 (25.0)	0 (0.0)	1 (14.3)	1 (14.3)	3 (42.9)	0 (0.0)	2 (28.6)
*S*. Typhimurium	1,4,5,12: i: 1,2 and 1,4,5,12: i: -	6 (21.4)	2(33.3)	0 (0.0)	0 (0.0)	0 (0.0)	0 (0.0)	5 (83.3)
*S*. Bredeney	1,4,12,27: l,v: 1,7	2 (7.1)	1 (50.0)	0 (0.0)	0 (0.0)	0 (0.0)	1 (50.0)	0 (0.0)
*S*. Enteritidis	1,9,12: g,m:	1 (3.6)	0 (0.0)	0 (0.0)	0 (0.0)	0 (0.0)	1 (100.0)	0 (0.0)
*S*. Urbana	30: b: e,n,x	1 (3.6)	0 (0.0)	1 (100.0)	0 (0.0)	0 (0.0)	0 (0.0)	0 (0.0)

N, total isolates; n = number in the group; % = percent, *S*. *enterica* subsp. *diarizonae* and *S*. Typhimurium have two antigen formula

### Virulence genes

**Fig 2:**
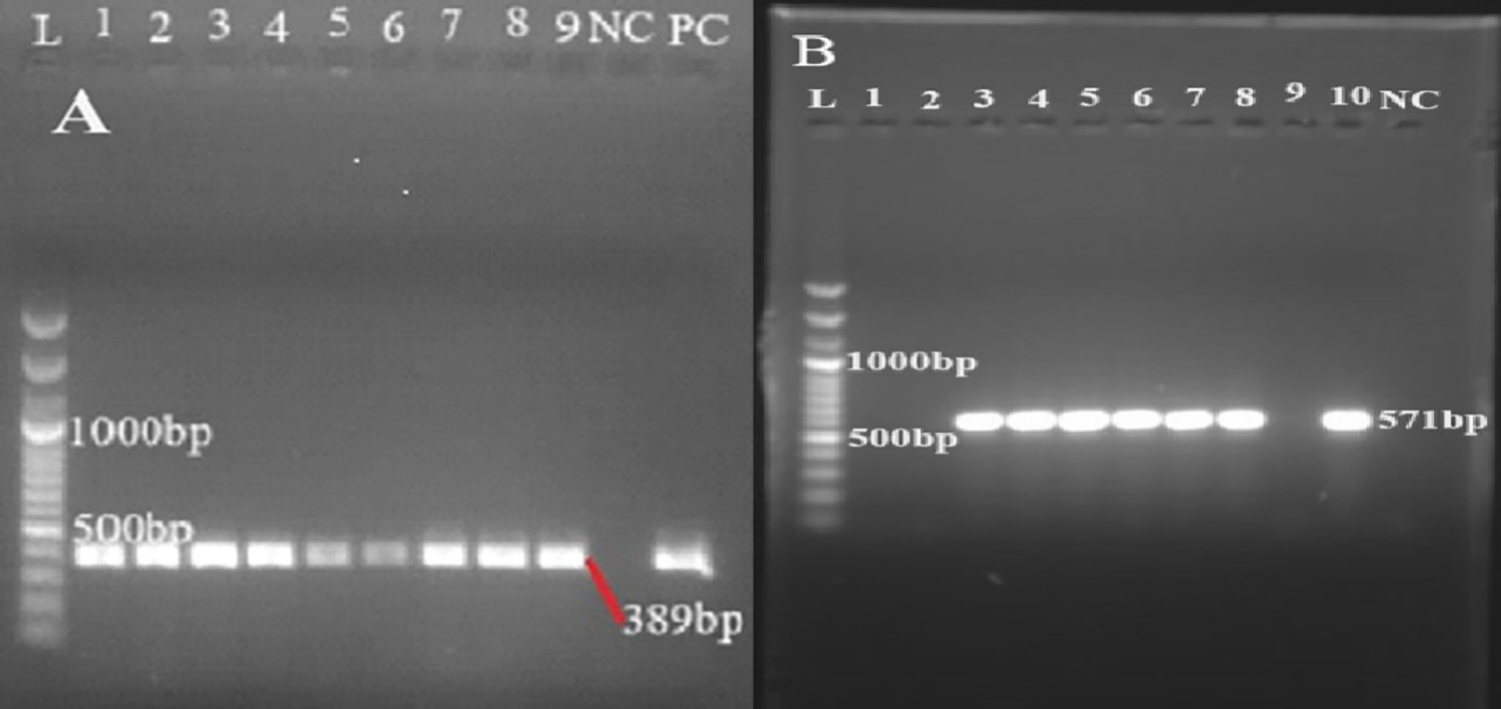
Representative two separate gel electrophoresis pictures of polymerase chain reaction (PCR) products (A. Gene *invA* fragments at 389 base pair (bp); Lanes 1–9, *invA* amplified from non-typhoidal *Salmonella* (NTS) isolates; B Gene *spv*C fragments at 571 bp, lanes 3,4,5,6,7, and 8 were amplified from NTS isolates. In all cases, lane L 100 bp molecular marker (ladder), lane PC or lane 10, positive control; Lane NC, negative control (S2 Fig).

In the current study, PCR amplification confirmed the presence of the two targeted virulence genes *(invA* and *spv*C*)* in different proportions. All 28 isolates contained the *invA* gene, whereas 21.4% of them were positive for the *spvC* gene. The *invA* was found in all types of samples, whereas the *spvC* gene was detected from milk, animal feces, and human stool samples. The gel pictures (PCR products) of *invA* and *spvC* genes are illustrated in Fig 2A and 2B, respectively. Table 5 shows the availability of virulence genes in each serovar. The *invA* gene was detected in all serovars whereas *spvC* was detected in *S*. Typhimurium and *S*. Enteritidis.

**Table 5:** Virulence gene detection in the serovars of the isolated.

Serovars	Number	Virulence genes		*MDR character (*n (%))
		*invA* (n (%))	*spvC* (n (%))	
*S*. Uganda	11	11 (100.0)	0 (0.0)	3 (27.3)
*S*. *enterica* subsp. *diarizonae*	7	7 (100.0)	0 (0.0)	4 (57.1)
*S*. Typhimurium	6	6 (100.0)	5 (83.3)	5 (83.3)
*S*. Bredeney	2	2 (100.0)	0 (0.0)	0 (0.0)
*S*. Enteritidis	1	1 (100.0)	1 (100.0)	0 (0.0)
*S*. Urbana	1	1 (100.0)	0 (0.0)	1 (100.0)

n = number, % = percent; MDR, Multidrug resistance.

### Antimicrobial resistance profile

**Fig 3:**
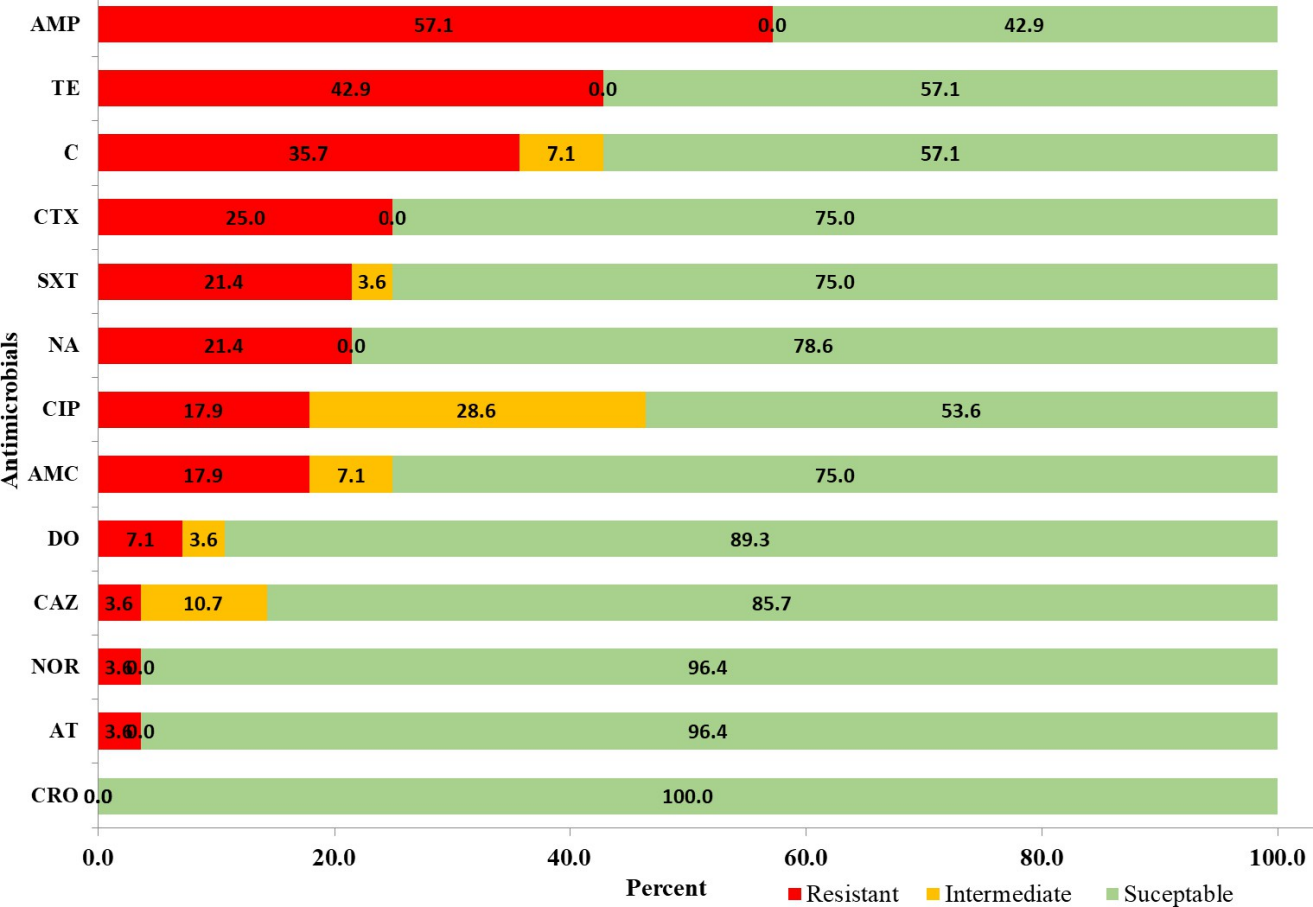
Antimicrobial susceptibility patterns of 28 *Salmonella* isolates. AMP, ampicillin; AMC, amoxicillin-clavulanic acid; CRO, ceftriaxone; CTX, cefotaxime; CAZ, ceftazidime; C, chloramphenicol; TE, tetracycline; DO, doxycycline; AT, azithromycin; NA, nalidixic acid; NOR, norfloxacin; CIP, ciprofloxacin; SXT, sulphamethoxazole-trimethoprim. Isolates were divided into susceptible, intermediate, or resistant based on CLSI guidelines [40] (S1 Data).

Non-typhoidal *Salmonella* isolates (n = 28) were tested against 13 commonly used antimicrobials representing 8 classes. The result showed that a higher proportion of resistance was observed in ampicillin (57.1%), tetracycline (42.9%), and chloramphenicol (35.7%). On the other hand, NTS isolates were more susceptible to ceftriaxone (100%), azithromycin (96.4%), and norfloxacin (96.4%) (Fig 3). Among the NTS isolates tested, 89.3% were resistant to one or more classes, and 46.4% were resistant to more than two classes of antimicrobial agents (multi-drug resistance (MDR)) [41, 42]. The result also showed that MDR isolates were isolated from animals, humans, milk, and the environment (Table 6). Table 6 also shows the resistance pattern of NTS isolates.

**Table 6:** Resistant pattern of 28 non-typhoidal *Salmonella*, and type of samples.

No of classes of antimicrobials*	No of Isolates (n (%))	Resistance pattern	No in the Style	Type of Samples (Number of isolates)
Resistant to none	3 (9.7)			Pooled raw milk (3)
Resistant to one	5 (16.1)	C	1	Human stool (1)
		TE	2	Dairy farm sewage (2)
		AMP	1	Dairy farm sewage (1)
		NA	1	Animal feces (1)
Resistant to two	7 (22.6)	AMP, CTX	2	Dairy farm sewage (1), Animal feces (1)
		AMP, TE	3	Milk container swabs (1), Dairy farm sewage (1) and Human stool (1)
		AT, NOR	1	Milk container swabs (1)
		TE, SXT	1	Pooled raw milk (1)
Resistant to three	7 (22.6)	AMC, C, CIP	1	Pooled raw milk (1)
		AMC, CIP, SXT	1	Animal feces (1)
		AMP, C, TE/DO	1	Animal feces (1)
		AMP, CTX, C	2	Animal feces (2)
		AMP, TE, CIP	1	Animal feces (1)
		TE, NA, CIP	1	Milk container swabs (1)
Resistant to four	3 (9.7)	AMP, C, NA, SXT	2	Animal feces (2)
		AMP, AMC, C, TE	1	Pooled raw milk (1)
Resistant to five	2 (8.5)	AMP, AMC, CTX, TE/DO, SXT	1	Milkers hand swabs (1)
		AMP, CTX, C, TE, NA	1	Dairy farm Sewage (1)
Resistant to six	1 (3.2)	AMP, AMC, CTX/CAZ, C, NA/CIP, SXT	1	Animal feces (1)

No, n = number; %, percent; AMP, ampicillin; AMC, amoxicillin-clavulanic acid; CRO, ceftriaxone; CTX, cefotaxime; CAZ, ceftazidime; C, chloramphenicol; TE, tetracycline; DO, doxycycline; AT, azithromycin; NA, nalidixic acid; NOR, norfloxacin; CIP, ciprofloxacin; SXT, sulphamethoxazole-trimethoprim (S1 Data)

## Discussion

Detection of Non-typhoidal *Salmonella* in the feces of animals, milk, milk containers, and farm environments can be potential sources for human infections and subsequent spreading to the public at large [43]. Without a thorough understanding of the potential reservoirs, it is practically impossible to design effective control and prevention strategies. In this study, we demonstrated that the main components of the human-dairy interface (human, animal, milk, and environment like sewage) are the reservoir and potential source of NTS infection.

The factors that make NTS survive and shed in apparently healthy humans and animals are not well exploited. However, pathogen-related factors like adherence and secretory effectors, other virulence factors, and host-related factors like immune response may play a role in the survival and shedding of the pathogen [44]. As it has been indicated in the results of this study, apparently healthy individuals can be the asymptomatic carriers of NTS and play a role in the fecal-oral transmission and contamination of food, especially by food handlers for future outbreaks. For control and prevention of NTS, these individuals can be a threat and require special attention [44]. The presence of NTS in the feces of apparently healthy animals is also an indication that feces of an animal can be the source of milk or environmental contamination which keeps the public at risk of NTS infection [45].

Bacteria like NTS enter into the milk mainly during and/or after milking from uncleaned teat or udder of the animal, the milker, the milking environment (dust), the water used to clean the milk containers, flies or other insects, the addition of contaminated water, or other adulteration practices [46]. The proportion of NTS in pooled raw milk was 10.3% in the current study. In line with this, a previous study showed that the prevalence of NTS in raw milk in Ethiopia was 10.8% [47]. However, as high as 19.4% prevalence of NTS in raw milk was also reported by Bedassa *et al*. [30]. The difference may be related to the factors that promote the survival and spread of the pathogen and the chance of contamination of the raw milk [30,43].

The NTS can be found not only in animals and food like milk but also in the environment (sewage). The results of this study showed that sewage can serve as a reservoir of NTS. The presence of the isolates in the sewage also showed their ability to survive in different environments. The pathogen in the sewage can contaminate water bodies like rivers, underground water, or vegetables if released without appropriate treatment [48]. People can acquire the bacteria by direct contact with the sewage with inadequate hand washing, drinking contaminated water, or eating contaminated vegetables [49].

Assessing and determining the risk factors of NTS is very essential for the control and prevention of the disease. In this study, even though different risk factors were considered, strong evidence regarding the influence of factors was not obtained. Hence, large-scale and longitudinal studies are necessary to understand the factors that may contribute to the presence of the pathogen in the human-dairy interface [50]. We detected NTS in all sampling sites. The presence of the pathogen in different regions of Ethiopia was also previously reported [30]. This may be related to the hygienic status of farms, the health of animals, and husbandry practices. These all showed that intervention measures should be conducted in the study areas and beyond.

Identifying the serovars of *Salmonella* is a very important practice since the severity of the disease, the geographic area, the animal affected, environmental adaptability, and antimicrobial resistance pattern may vary among serovars [16,35]. The findings of this study showed that different serovars of NTS were circulating in the human-dairy interface, which necessitates the designing of control and prevention strategies. These serovars have been reported not only in the community and dairy farms but also in healthcare facilities and clinical cases since they were reported by Kebede *et al*. [51] and Amare *et al*. [52].

The pathogenicity of NTS is mainly related to the presence of virulence factors [53–55]. The virulence factors may be found on mobile genetic elements (plasmids, transposons) and specific sections of the bacterial chromosome, like pathogenicity islands [56,57]. In this study, the presence of the two common virulence genes (*invA* and *spvC*) encoding different virulent factors was investigated. The *invA* gene helps the bacterium to invade and penetrate the host cells. In this study, the *invA* gene was detected in all isolates, and this is supported by a previous report that showed a 100% detection of the *invA* gene among NTS isolates in Malaysia [12], Iran [58], and Egypt [19]. Detection of the *invA* gene among NTS isolates was recommended for the diagnosis of the pathogen since it occurs in all strains [59]. The *spvC* gene enables the pathogen to survive within the macrophages and facilitate systemic infections [60]. In this study, 21.4% of the isolates were positive for the *spvC* gene. Previously, 30.6% *spvC* gene prevalence among NTS isolates was reported in Egypt [19] and 37% prevalence was also reported in Iran [58].

The emergence and spread of AMR in pathogens like NTS is an important challenge in the world, particularly in low-income countries where the prevalence of bacterial pathogens in general and NTS, in particular, is high. In this study, 46.4% were MDR. The MDR strains are usually associated with higher morbidity (frequent bloodstream infections and hospitalizations) and mortality than susceptible strains [61,62]. In Ethiopia, the problem is frequently reported [63]; the study conducted by Beyene *et al*. [64] and Tweldemedhin *et al*. [65] reported that the prevalence of MDR among *Salmonella enterica* isolates was 59.46% and 65%, respectively. Nowadays, due to the emergence of resistance strains, the treatment regimen for *Salmonella* infection is shifted to fluoroquinolones and third-generation cephalosporins [52]. However, increasing the burden of AMR, resistant strains to these preferred antimicrobials have also been emerging.

In this study, resistant strains were detected in humans, animals, food (milk), and the environment (sewage), which provided further evidence that AMR is not only an issue of human and animal health sectors but also the environment. Park *et al*. [66] and Beyene *et al*. [67] reported that environmental waste (slurry) from dairy was one source of AMR and a critical point for the mitigation of AMR.

### Limitations

In this study, dairy farms were visited once, and numerators (positives) were small, which didn’t allow us to generate strong evidence to see the influence of factors like seasons, farm practices, and others. Hence, long-term study and repeated sampling were preferable for a better understanding of the influence of factors.

## Conclusions

The study provided valuable information regarding NTS in the human-dairy interface. The bacterium was detected in considerable proportion in Northwest Ethiopia and all types of samples (cow’s feces, pooled raw milk, milk container, human stool, hand swabs, and dairy farm sewage) and sampling sites. The farm level detection was high, which demonstrates NTS is circulating in the human-dairy interface. Six serovars that belong to two subspecies were detected. The two common virulence genes (*invA* and *spv*C) were detected with different proportions. The NTS isolates were more resistant to ampicillin, tetracycline, and chloramphenicol. However, the isolates were more susceptible to ceftriaxone, norfloxacin, and azithromycin. Intervention measures like hygiene, treating raw milk by appropriate pasteurization or boiling before consumption, and prudent use of antimicrobials have to be enhanced in dairy farms.

## Supporting information

S1 Data Raw data and summaries. (XLSX)

S1 Table Antimicrobial susceptibility testing performance standards for *Salmonella*. (DOCX)

S1 Fig Representative biochemical test results and plates. (PDF)

S2 Fig Gel pictures (PCR products) of the two target genes. (PDF)

S3 Fig A pictorial description of steps followed to isolate non-typhoidal from different samples. (PDF)
