# Supplementary material for: Serovars, virulence factors, and antimicrobial resistance profile of non-typhoidal Salmonella in the human-dairy interface in Northwest Ethiopia: A one health approach
Source: PLoS Negl Trop Dis. 2024 Nov 20;18(11):e0012646. doi: 10.1371/journal.pntd.0012646 (PMC11578527; doi:10.1371/journal.pntd.0012646)
Supplement: S1 Table — (DOCX) [file pntd.0012646.s002.DOCX]

Table 1. Antimicrobial susceptibility testing performance standards for *Salmonella*

| **S/N** | ***Group*** | ***Antimicrobials*** | ***Concentration*** | ***Code*** | ***Resistance*** | ***Intermediate*** | ***Susceptible*** |
| --- | --- | --- | --- | --- | --- | --- | --- |
|  | Beta-lactam Penicillins | Ampicillin | 10µg | AMP | ≤13 | 14-16 | ≥17mm |
|  | Beta-lactam combination agents | Amoxicillin-clavulanic acid | 20/10 µg | AMC | ≤13 | 14-17 | ≥18mm |
|  | Beta-lactam cephalosporin first-generation | Cefalotin | 30 µg | KF | ≤14 | 15-21 | ≥22mm |
|  | Beta-lactam cephalosporin secondary generation | Cefoxitin | 30 µg | CX | ≤14 | 15-17 | ≥18mm |
|  | Beta-lactam cephalosporin third generation | Ceftazidime | 30 µg | CAZ | ≤17 | 18-20 | ≥21mm |
|  |  | Ceftriaxone | 30 µg | CRO | ≤19 | 20-22 | ≥23mm |
|  |  | Cefotaxime | 30 µg | CTX | ≤22 | 23-25 | ≥26mm |
|  | Phenols | Chloramphenicol | 30 µg | C | ≤12 | 13-17 | ≥18mm |
|  | Tetracyclines | Tetracycline | 30 µg | TE | ≤11 | 12-14 | ≥15mm |
|  |  | Doxycycline | 30 µg | DO | ≤10 | 11-13 | ≥14mm |
|  | Macrolides | Erythromycin | 15 µg | E | ≤13 | 14-21 | ≥22mm |
|  |  | Azithromycin | 15 µg | AZM | ≤12 | - | ≥13mm |
|  | Aminoglycosides | Gentamicin | 10 µg | CN | ≤14 | 15-17 | ≥18mm |
|  |  | Kanamycin | 30 µg | K | ≤13 | 14-17 | ≥18mm |
|  | Fluoroquinolones | Nalidixic acid | 30 µg | NA | ≤13 | 14-18 | ≥19mm |
|  |  | Norfloxacin | 10 µg | NOR | ≤12 | 13-16 | ≥17mm |
|  |  | Ciprofloxacin | 5 µg | CIP | ≤20 | 21-30 | ≥31mm |
|  | Folate pathway antagonists | Sulphamethoxazole-trimethoprim | 23.75/1.25 µg | STX | ≤10 | 11-15 | ≥16mm |

Source**:** Clinical and laboratory standard institute**:** Performance Standards for Antimicrobial Susceptibility Testing; Twenty-Third Informational Supplement. 2023. [40]
