# Supplementary material for: Serovars, virulence factors, and antimicrobial resistance profile of non-typhoidal Salmonella in the human-dairy interface in Northwest Ethiopia: A one health approach
Source: PLoS Negl Trop Dis. 2024 Nov 20;18(11):e0012646. doi: 10.1371/journal.pntd.0012646 (PMC11578527; doi:10.1371/journal.pntd.0012646)
Supplement: S1 Fig — (PDF) [file pntd.0012646.s003.pdf]

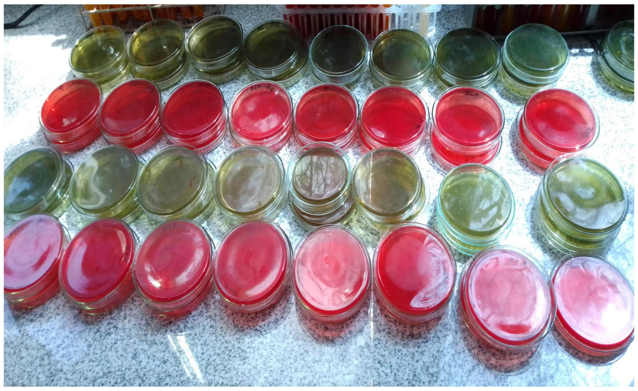

A. XLD (red), and HE (green) agars prepared for inoculation

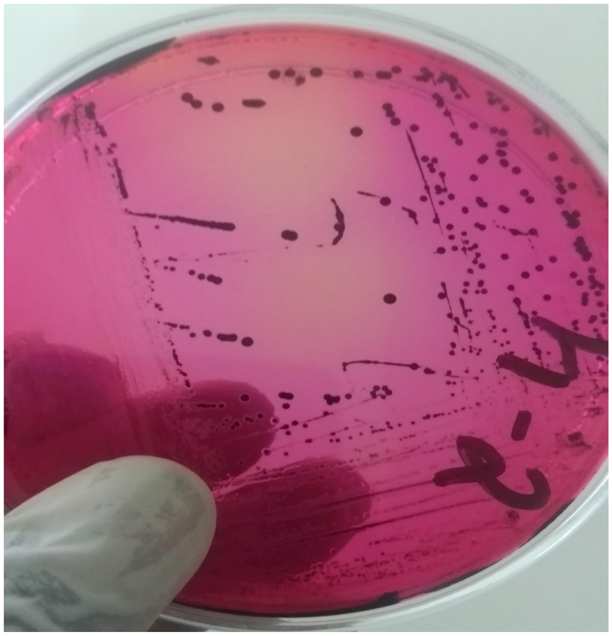

B. Suspected Salmonella colonies on XLD agar

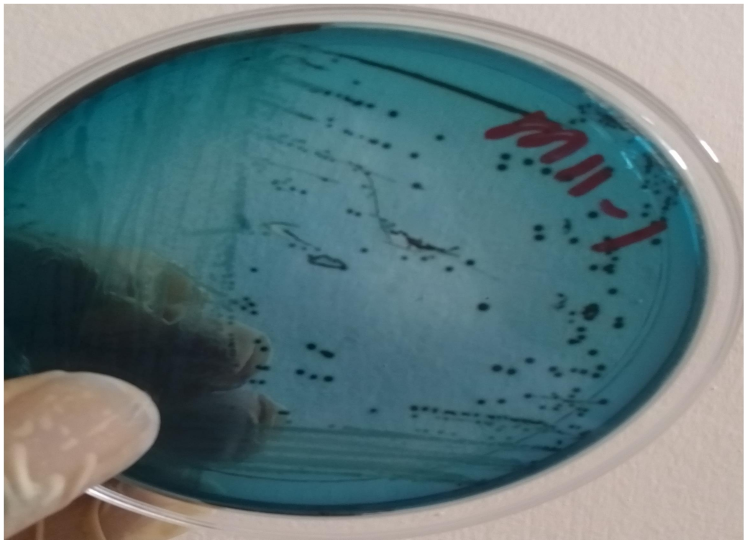

C. Suspected Salmonella colonies on HE agar

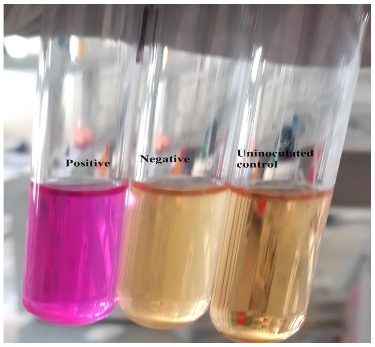

D. Urease production test, *Salmonella* is urease negative

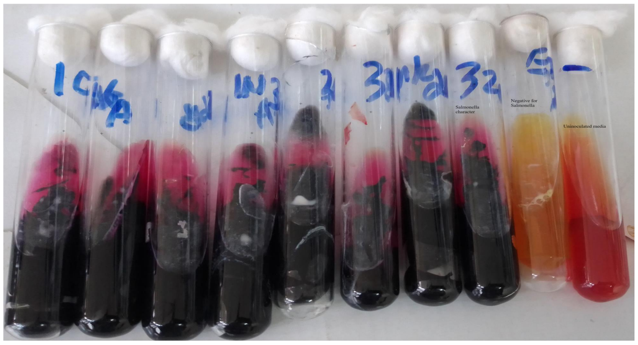

E. Triple sugar iron test (TSI) black and red tubes are character of Salmonella

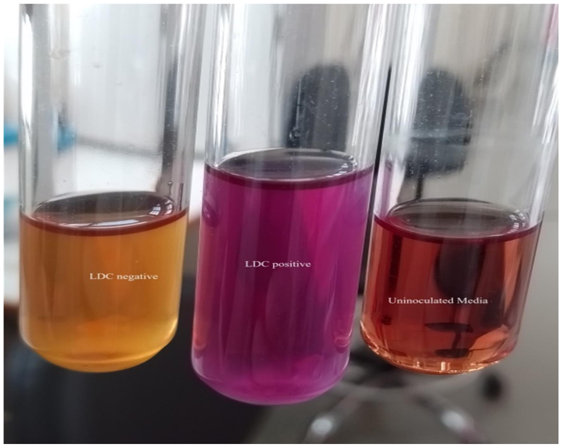

F. Lysine decarboxylase test (LDC) Salmonella is LSD positive

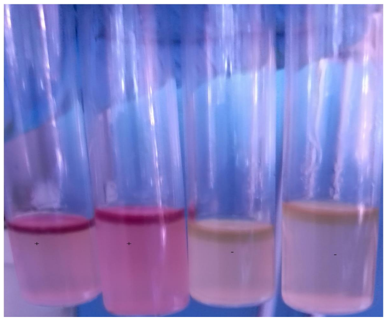

G. Indole test (Most Salmonella Strains are indole negative)
