# Supplementary figures and images for: Serovars, virulence factors, and antimicrobial resistance profile of non-typhoidal Salmonella in the human-dairy interface in Northwest Ethiopia: A one health approach
Source: PLoS Negl Trop Dis. 2024 Nov 20;18(11):e0012646. doi: 10.1371/journal.pntd.0012646 (PMC11578527; doi:10.1371/journal.pntd.0012646)

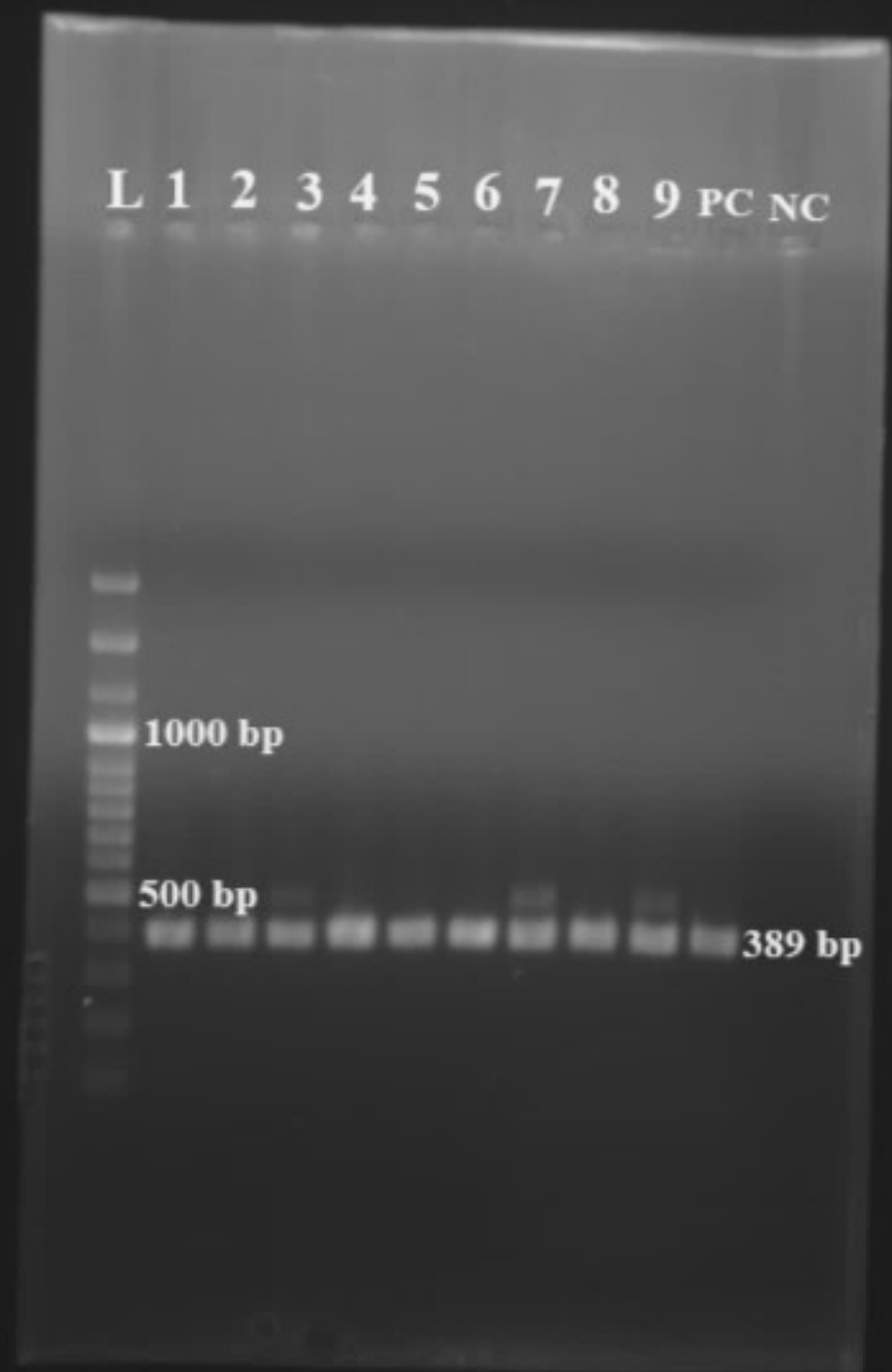

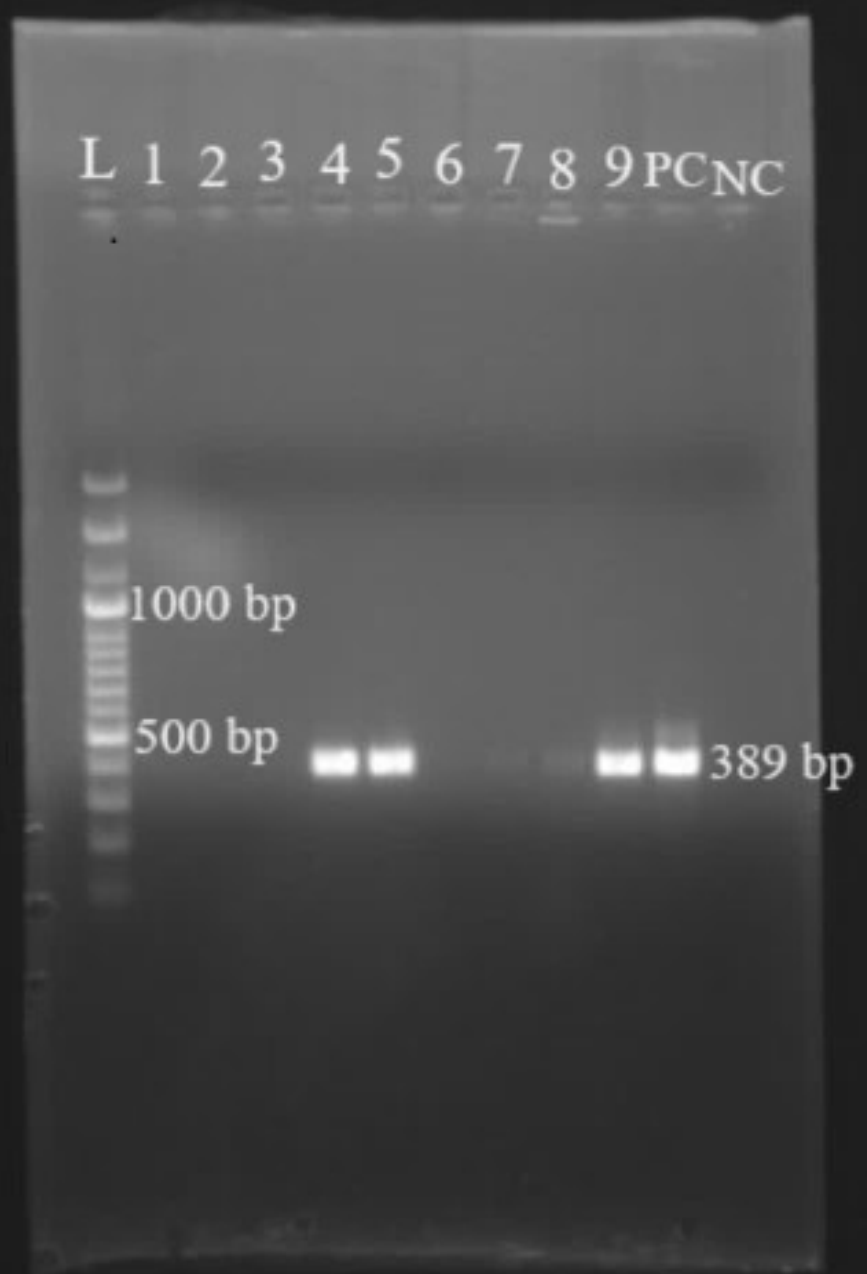

L 1 2 3 4 5 6 7 8 9 PCNC

1000 bp  
800 bp  
600 bp  
500 bp  
400 bp  
300 bp  
200 bp  
100 bp

500 bp

389 bp

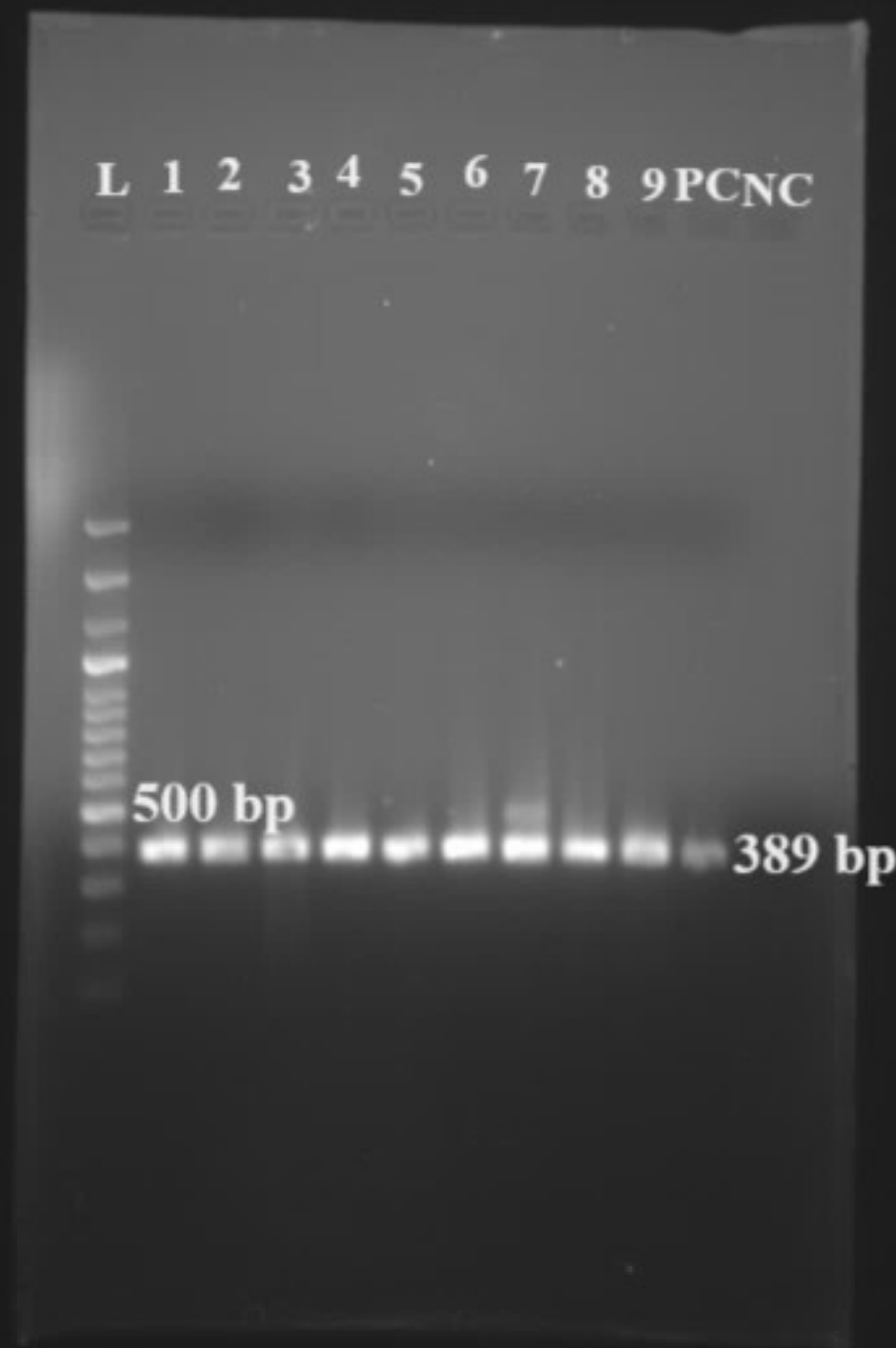

NC PC 7 6 5 4 3 2 1 L

389 bp

1000 bp

500 bp

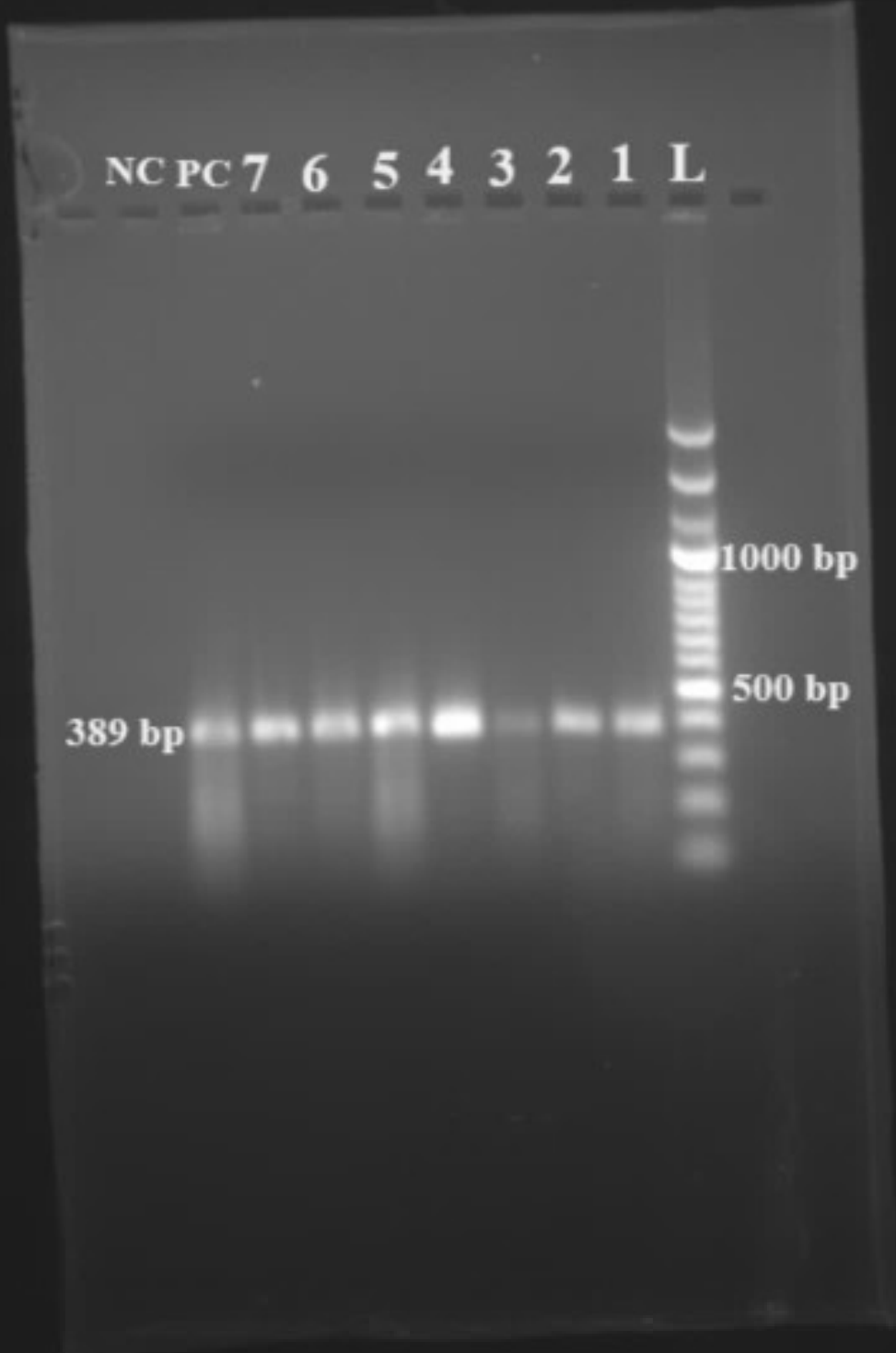

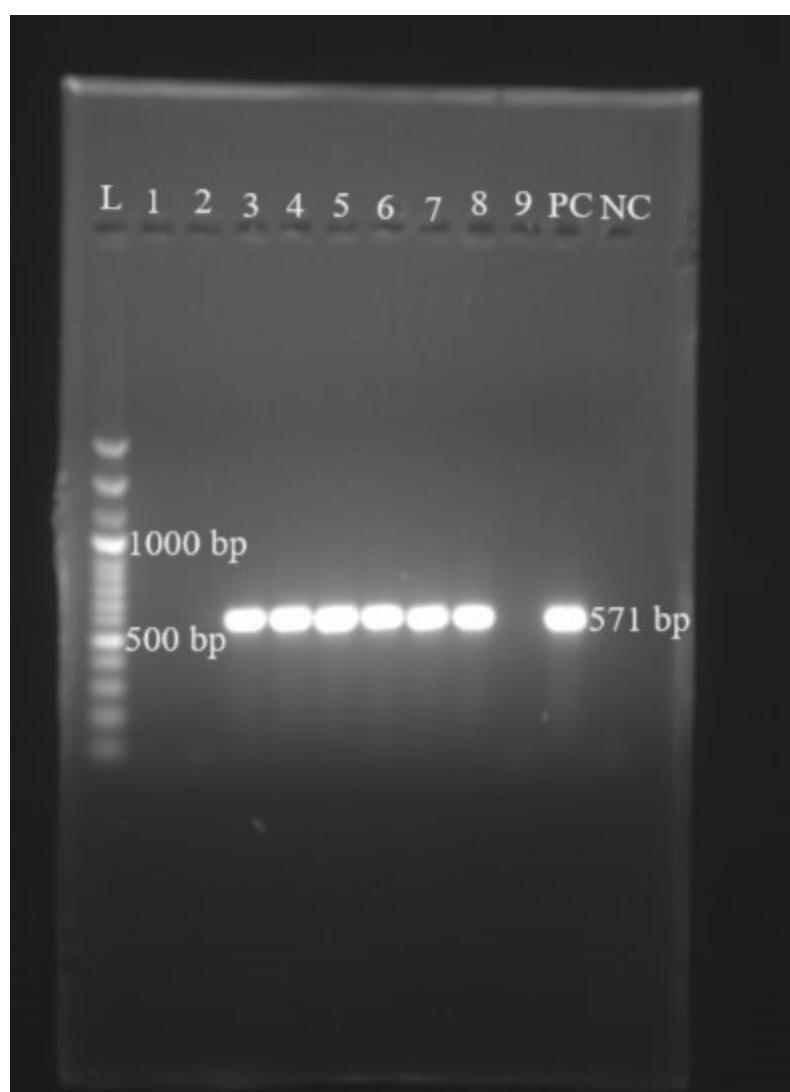

### Interperetation

[illegible]

Supplement: S2 Fig — (PDF) [file pntd.0012646.s004.pdf]

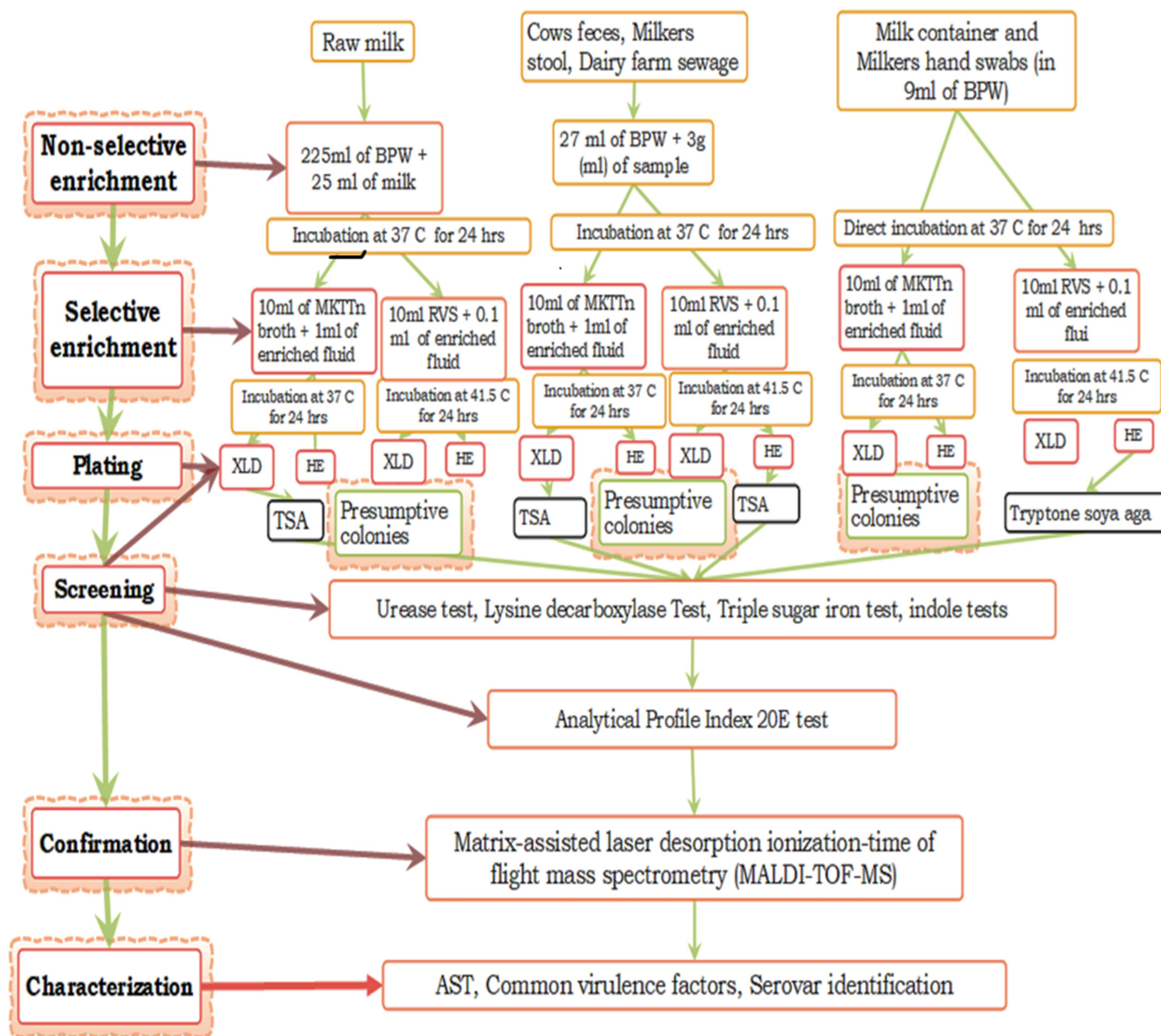

Supplement: S3 Fig — (PDF) [file pntd.0012646.s005.pdf]
